# Pectin Enhances Bio-Control Efficacy by Inducing Colonization and Secretion of Secondary Metabolites by *Bacillus amyloliquefaciens* SQY 162 in the Rhizosphere of Tobacco

**DOI:** 10.1371/journal.pone.0127418

**Published:** 2015-05-21

**Authors:** Kai Wu, Zhiying Fang, Rong Guo, Bin Pan, Wen Shi, Saifei Yuan, Huilin Guan, Ming Gong, Biao Shen, Qirong Shen

**Affiliations:** 1 National Engineering Research Center for Organic-based Fertilizers, College of Resources and Environmental Sciences, Nanjing Agricultural University, Nanjing, 210095, China; 2 Engineering Research Center of Sustainable Development and Utilization of Biomass Energy, Key Laboratory of Ministry of Education, College of Energy and Environmental Sciences, Yunnan Normal University, Kunming, 650500, China; 3 School of Life Sciences, Yunnan Normal University, Kunming, 650500, China; Leibniz-Institute of Vegetable and Ornamental Crops, GERMANY

## Abstract

*Bacillus amyloliquefaciens* is a plant-beneficial Gram-positive bacterium involved in suppressing soil-borne pathogens through the secretion of secondary metabolites and high rhizosphere competence. Biofilm formation is regarded as a prerequisite for high rhizosphere competence. In this work, we show that plant extracts affect the chemotaxis and biofilm formation of *B*. *amyloliquefaciens* SQY 162 (SQY 162). All carbohydrates tested induced the chemotaxis and biofilm formation of the SQY 162 strain; however, the bacterial growth rate was not influenced by the addition of carbohydrates. A strong chemotactic response and biofilm formation of SQY 162 were both induced by pectin through stimulation of surfactin synthesis and transcriptional expression of biofilm formation related matrix genes. These results suggested that pectin might serve as an environmental factor in the stimulation of the biofilm formation of SQY 162. Furthermore, in pot experiments the surfactin production and the population of SQY 162 in the rhizosphere significantly increased with the addition of sucrose or pectin, whereas the abundance of the bacterial pathogen *Ralstonia* decreased. With increased production of secondary metabolites in the rhizosphere of tobacco by SQY 162 and improved colonization density of SQY 162 in the pectin treatment, the disease incidences of bacterial wilt were efficiently suppressed. The present study revealed that certain plant extracts might serve as energy sources or environmental cues for SQY 162 to enhance the population density on tobacco root and bio-control efficacy of tobacco bacterial wilt.

## Introduction

The use of plant growth promoting rhizobacteria (PGPR) to bio-control soil-borne diseases and promote plant growth is a promising way to improve agriculture sustainability [1–2]. One PGPR example, the Gram-positive bacteria *Bacillus*, is widely used as biofertilizer to control *fusarium* and bacterial wilt [3–5]. *Bacillus amyloliquefaciens* produces lipopeptides (LPs) to protect the plant from pathogens [6]. Furthermore, *B*. *amyloliquefaciens* also induces the systemic resistance in plants against pathogens [7–9].

Successful root colonization of bio-control agents in the rhizosphere was essential for bio-control efficacy [4,9–10]. Chowdhury et al. [10] demonstrated that successful control of lettuce bottom rot was achieved through high rhizosphere competence of *B*. *amyloliquefaciens* FZB42 in the field. Additionally, the bio-control of *Fusarium oxysporum* on cucumber was facilitated by the biofilm formation of *B*. *amyloliquefaciens* on cucumber roots [11]. Biofilms are complex architectures in which numerous cells are embedded within a matrix consisting of exopolysaccharides (EPS), DNA and proteins [12–13]. The extracellular matrix was affected by the expression of the *epsA-O* operon and *tasA*, which are responsible for the synthesis of EPS and amyloid-like fibers, respectively [12,14]. *tasA* is indirectly controlled by the global transcriptional regulator Spo0A, which is controlled by various histidine kinases (KinA-KinE) under different environmental conditions [15–16]. Therefore, when appropriate environmental cues are present, the kinases are active and induce biofilm formation and root colonization.

Plants change the rhizobacterial living habitat through the secretion of root exudates, which act as nutrient sources or signal compounds in the rhizosphere [17–18]. Chen et al. [19] illustrated that tomato root exudates induced the biofilm formation of *B*. *subtilis* 3610 via a KinD-dependent pathway. Tan et al. [20] demonstrated that the concentration of organic acids from root exudates from different tomato cultivars affected the biofilm formation and rhizosphere colonization of *B*. *amyloliquefaciens* T-5. Furthermore, structural carbohydrates from *Arabidopsis* acted as a more important factor than the root exudates in triggering the biofilm formation and root colonization of *B*. *subtilis* [15].

Surfactin secreted from *B*. *amyloliquefaciens* may be important in biofilm formation, root colonization, plant defense stimulation and the effective suppression of plant disease [7,21–23]. Recently, our study found that *B*. *amyloliquefaciens* SQY 162 (SQY 162) could suppress tobacco bacterial wilt caused by *Ralstonia solanacearum* through changing the bacterial community composition in rhizosphere [24]. However, interaction between the bio-control activity of the strain SQY 162 and the plant polysaccharides is unknown. Therefore, we hypothesized that plant polysaccharides could improve the colonization of SQY 162 and bio-control activity against tobacco bacterial wilt due to enhancing surfactin production and reducing the population density of the pathogen. The effects of plant root extracts and different carbohydrates on the chemotaxis and biofilm formations of strain SQY 162 were identified *in vitro* with capillary and microtitre plate assay, respectively. The expression analyses of several genes involved in the production of the matrix and surfactin of strain SQY 162 were performed in defined medium with carbohydrates *in vitro* and pot experiments were used to analyze the disease incidences. The *Bacillus*-tobacco plant model system could be useful for understanding the bacteria-plant interactions in future plant protection studies.

## Materials and Methods

### Ethics statement

The *B*. *amyloliquefaciens* strain SQY 162 was isolated from the soil from the farmland (26° 74′ N, 107° 49′ E) in Guizhou, China, with property rights. We collaborate with the Institute of Guizhou Tobacco Science Research on the study about biological control of tobacco bacterial wilt. Therefore, we have obtained permission from the chairman of the institute. The chairman of the institute, Yonggang Feng, should be contacted for future permission. The locations are not protected, and the field studies did not involve endangered or protected species.

### Strain

*B*. *amyloliquefaciens* SQY 162 (SQY162, CGMCC accession No.7500, China General Microbiology Culture Collection Center) was isolated from the rhizosphere soil in a diseased field in Fuquan, China and showed strong antagonistic ability against the tobacco bacterial wilt pathogen *Ralstonia solanacearum in vitro* (S1 Fig) [24]. SQY 162 was spotted on the nutrient medium (for 1 L liquid nutrient medium containing 3 g beef extracts, 10 g tryptone, 10 g NaCl) with sterile toothpicks. The beef extracts and tryptone were purchased from Oxoid Ltd., Basingstoke, UK. After 24 h incubation, the plates were sprayed with cell suspension of *R*. *solanacearum* (nearly 10^7^ cfu/ml) and then incubated for an additional 24h to observe the inhibition activity.

*R*. *solanacearum* with its strong pathogenicity was isolated from diseased tobacco plants in Sansui as described by Wu et al. [24]. The strain was grown in Casamino acid-Peptone-Glucose (CPG) medium (Oxoid Ltd., Basingstoke, UK) [25]. If necessary, antibiotics were used in following concentration: ampicillin, 100 mg/L; kanamycin, 25 mg/L; tetracycline, 15 mg/L; and gentamicin, 12.5 mg/L. Antibiotics were purchased from Sigma-Aldrich (St Louis, Mo, USA).

### Plant root exudates and root extracts preparation

Tobacco seeds were surface-sterilized and sown in floating polystyrene trays. Tobacco plants cultured for 30 days were used for root exudate collection. The root exudates collection procedure was conducted as described previously by Hao et al. [26] with a few modifications. Briefly, plants were gently washed three times with sterilized double-distilled water and then transplanted in plastic cups containing 300 ml sterilized double-distilled water. Each treatment was conducted in triplicate (each cup consisted of three tobacco plants). After incubation in the plant growth chamber at 28°C for 24 h (16 h light/8 h dark), root exudates were collected, lyophilized and sterilized by passing through a 0.2-μm filter, and stored at -80°C for further analysis.

For plant root extracts, the plant roots were frozen with liquid nitrogen and ground with mortar and pestle, re-suspended in 100 ml sterile water, lyophilized and filtered with a 0.2-μm filter, and stored at -80°C for further analysis [15].

### Chemotaxis experiments

The capillary assay was performed according to the previously published methods with several modifications [27]. The SQY 162 strain was grown in 3 ml Luria-Bertani (LB) broth (Oxoid Ltd., Basingstoke, UK) overnight. Then, 1 ml liquid culture was transferred to 100 ml fresh LB broth with 170 r/min shaking at 30°C until the OD_600_ reached 0.3 to 0.7. Cells were then collected by centrifugation, washed twice in sterile phosphate buffer (10 mM potassium phosphate, pH 7.0, 0.1 mM EDTA, and 1 mM MgSO_4_) [28], and diluted to an OD_600_ of 0.1 for future testing. A 200-μl pipette tip was used as a chamber to incubate 100 μl SQY 162 suspended in phosphate buffer and harvested as described above. A 4 cm-25-gauge needle (Becton-Dickinson) was used as the capillary for chemotaxis and was attached to a 1 ml tuberculin syringe containing 100 μl of the 15 × root exudates or plant root extracts or different types of compounds. After 2 h incubation at 30°C in the incubation chamber, the needle syringe was removed and the contents were serially diluted in sterile phosphate buffer and plated on LB medium. The bacterial number in the capillaries was calculated as the average from the colony forming units (CFU) ml^-1^ counted in triplicate plates. Each treatment was presented with three separate capillary assays.

### Biofilm formation assay

Biofilm formation was quantified with a modified version of the polyvinylchloride (PVC) microtitre plate assay as described previously by Hamon and Lazazzera [29]. The cells of strain SQY 162, growing in LB medium to the mid-exponential growth stage, were collected and diluted to an OD_600_ of 0.01 in LB medium. SQY 162 suspensions (100 μl) containing the 15 × tobacco root exudates, root extracts or different types of compounds were then added to each well of a 96-well PVC microtiter plate. The inoculated plates were incubated under stationary conditions at 30°C for 18 h. Medium and non-adherent cells were removed, and adherent cells were washed twice with phosphate buffer, as described above, and air-dried. Adherent cells were then stained with 1% crystal violet (CV) in phosphate buffer at room temperature for 20 min. Excess CV was then removed, and the wells were rinsed with water. The bound CV was solubilized in 200 μl of 80% ethanol and 20% acetone. Biofilm formation was then measured at A_570_ for each well.

### Effects of carbohydrates on the growth of SQY 162 and *R*. *solanacearum*

The cells of SQY 162 were incubated overnight in 3 ml LB medium as described above. One milliliter SQY 162 liquid culture was inoculated into 100 ml LB medium containing carbohydrates at a final concentration of 0.5% (w:v). For *R*. *solanacearum*, the pathogen strain was incubated for 48 h in 3 ml nutrient medium as described above. One milliliter *R*. *solanacearum* liquid culture was inoculated into 100 ml nutrient medium containing carbohydrates at a final concentration of 0.5% (w:v). The cultures were then incubated for 60 h at 30°C with 170 rpm shaking. Cultures were measured at OD_600_ for each treatment.

### Effects of carbohydrates on the lipopeptide production of SQY 162

The cells were incubated overnight in 3 ml LB medium. One milliliter SQY 162 liquid culture was inoculated into 100 ml LB medium containing compounds at a final concentration of 0.5% (w:v). The cultures were then incubated for 60 h at 30°C with 170 rpm shaking. To isolate lipopeptide products, the liquid medium was centrifuged at 10,000 rpm at 4°C, and 6 mol/L HCl was added into the supernatant to a final pH of 2.0 to precipitate the crude lipopeptides. The precipitates were than stored at 4°C overnight. The crude LPs were harvested by 10,000 rpm centrifugation for 10 min at 4°C and then extracted with methanol. The solutions were evaporated with a rotary evaporator, and the resulting residues were re-suspended in distilled water and lyophilized at -80°C. The lyophilized compounds were separated into two parts, one for HPLC analysis and the other for calculation of crude LPs production. The total amounts of crude LPs were calculated as the average value of three replicates.

### HPLC analysis

The crude LPs were dissolved in methanol and passed through a 0.22 μm filter. HPLC analysis using an XDB-C18 column (4.6 mm × 250 mm, Agilent Technologies, Santa Clara, CA, USA) was performed at 30°C with the mobile phase consisting of 10 mM trifluoroacetic acid (TFA):acetonitrile (30:70,for surfactin; 60:40, for iturin A, v:v) buffer solution. Each sample solution (10 μl) was eluted for 10 min at a flow rate of 0.8 ml min^-1^ and detected at 280 nm. Standards for surfactin and iturin A were purchased from Sigma-Aldrich (St Louis, MO,USA) and chromatographed alone.

### Pot experiment

The effects of SQY162 plus carbohydrates on the control of tobacco bacterial wilt were evaluated in a green house. Cells of SQY162 were cultured in LB media overnight. The cells were harvested by centrifugation and washed twice with sterile phosphate buffer (10 mM potassium phosphate, pH 7.0, 0.1 mM EDTA, and 1 mM MgSO_4_) [28] and adjusted to a cell density of 1.5 × 10^9^/ml. The sandy loam soil (pH 5.31, Organic Carbon 12.37 g/Kg, total N 1.74 g/Kg, and available K, P contents 214.26, 53.81 mg/Kg, respectively) collected from a diseased field in Sansui County in Guizhou province was steam-sterilized twice at 121°C for 40 min. For the preparation of cell suspension of *R*. *solanacearum*, the overnight liquid culture of *R*. *solanacearum* in CPG broth as described above was centrifuged and obtained cells were suspended in sterile phosphate buffer (3.0 × 10^8^/ml). The steam-sterilized soil was inoculated with *R*. *solanacearum* at a final concentration of 10^6^ cfu/g of soil. The numbers of *R*. *solanacearum* were determined using semi-selective medium from South Africa (Oxoid Ltd., Basingstoke, UK) composed of peptone 10 g, glycerol 5 ml, casamino acid 1 g, agar 20 g; crystal violet 5 mg; polymyxin B sulfate 100 mg; bacitracin 25 mg; chloromycetin 5 mg; penicillin 0.5 mg; and cycloheximide 100 mg [30]. Antibiotics were purchased from Sigma-Aldrich (St Louis, Mo, USA). Surface-sterilized tobacco seeds (cultivar K326, provided by the Institute of Guizhou Tobacco Science Research) were sown in floating polystyrene trays. After growing for 7 weeks, seedlings were washed three times with sterile water and then transferred to plastic cups (700-g soil). The treatments were designed as follows: (1) Control, soil treated with nothing; (2) PC, yeast extract at a final concentration of 1% was inoculated into each cup; (3) Su, sucrose at a final concentration of 1% was inoculated into each cup; and (4) Pectin, pectin at a final concentration of 1% was inoculated into each cup. Each sample was inoculated with SQY 162 at a final concentration of 10^7^ cfu/g of soil before tobacco transplanting and was performed with three replicates. Each replicate consisted of 5 cups (one tobacco seedling was transplanted in each cup). All the plants were placed in a green house (16 h light/8 h dark, 28–32°C). The disease incidence was calculated as the percentage of diseased plants to the total number of transplanted plants in each replicate. Thirty days after transplanting (in the fast growing stage of tobacco), ten-gram rhizosphere soil from the pot was extracted with 40 ml sterile water and was centrifuged. The supernatant was collected for further crude LPs determination. Crude LPs containing surfactin and iturin in the supernatant were extracted and analyzed according to the procedures described above.

The populations of SQY 162 were counted with plate counting methods at 30 days after transplanting. The population of SQY 162 was counted on selective medium supplemented with polymyxin B (35 μg/ml), lincomycin (5 μg/ml) and cycloheximide (50 μg/ml) [24]. The number of *R*. *solanacearum* was determined using semi-selective medium from South Africa as described above.

### Transcription analysis of genes involved in biofilm formation

Biofilm of strain SQY 162 formed under stationary conditions at 30°C for 18 h was harvested, and RNA from SQY 162 was extracted according to the procedure described by Xu et al. [31]. Briefly, after biofilm formation at 30°C for 18 h, the cells from the sucrose and pectin treatments described above were centrifuged at 11000 rpm for 2 min (4°C). Total RNA was extracted using a Bacterial RNA Kit (Omega Bio-Tek, Inc., Norcross, GA, USA) according to the manufacturer’s protocol. RNA samples were then reverse transcribed into cDNA in a 20 μl reverse transcription system (TransGen Biotech, Beijing, China) according to the manufacturer’s protocol.

Transcription levels of *epsD*, *yqxM*, *sft*, and *kinC* of SQY 162 cells from the different carbohydrate treatments were measured relative to SQY 162 (control) using quantitative reverse transcription-PCR (qRT-PCR) with a SYBR Premix Ex Taq (Perfect Real Time) Kit (TaKaRa). *epsD* (from the epsA-O operon) and *yqxM* (from the yqxM-sipW-tasA operon) are two genes critical in the production of exopolysaccharide (EPS) and TasA, respectively, which are essential for biofilm formation [31]. The *recA* gene was used as a control. qRT-PCR was performed with an ABI 7500 system under the following conditions: cDNA was denatured for 10 s at 95°C, followed by 40 cycles of 5 s at 95°C and 34 s at 60°C. The data of qRT-PCR were analyzed according to the method of 2^ˉ△△CT^ described by Livark and Schmittgen [32].

### Statistical analysis

Differences among treatments were assessed with ANOVA, and the means were analyzed with Microsoft Excel 2007 and then subjected to Duncan’s multiple range tests at P <0.05 using SPSS 13.0 statistical software (SPSS Inc., Chicago, USA).

## Results

### Chemotaxis response of SQY 162

**Fig 1:**
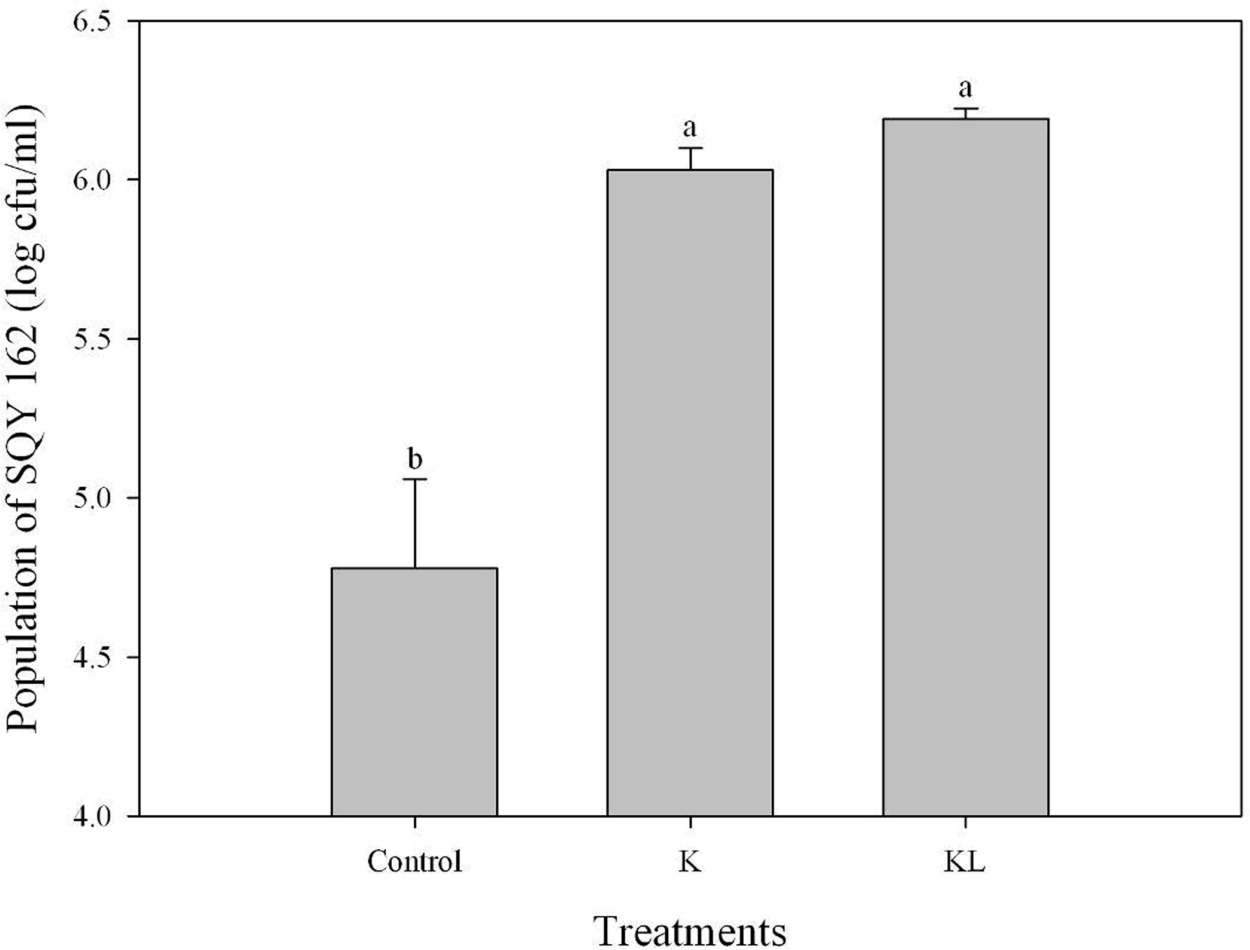
Chemotactic response of SQY 162 towards tobacco root exudates and root extracts from K326 evaluated by capillary assay. Control (chemotaxis buffer), K (inoculated with 15 × root exudates from K326), KL (inoculated with 15 × root extracts from K326).

**Fig 2:**
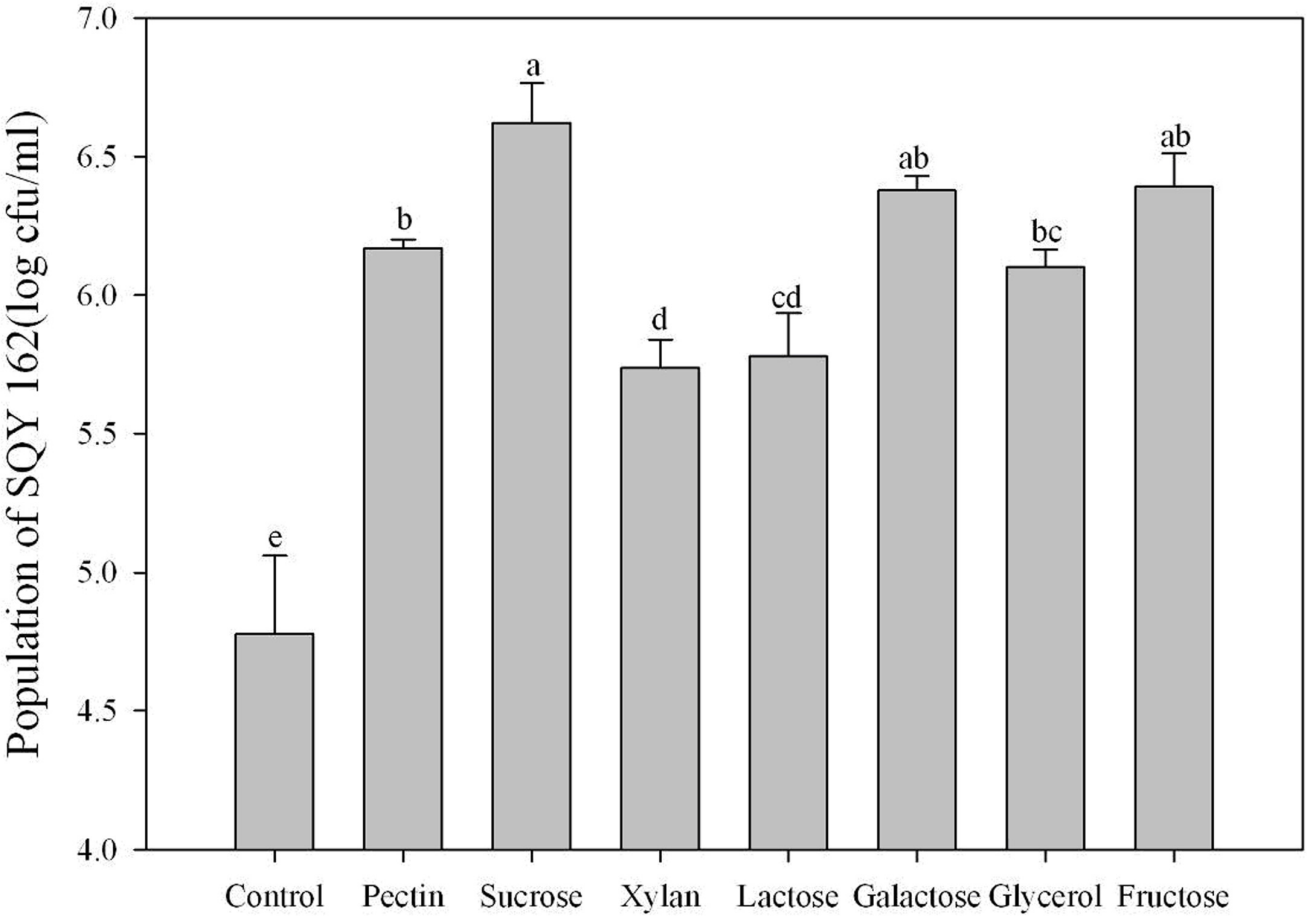
The chemotactic response of SQY 162 to treatment with different carbohydrates. The means and standard errors are shown. The different letters above each bar refer to the Duncan’s test, *p* < 0.05. Phosphate buffer was amended with sucrose (Sucrose), fructose (Fructose), pectin (Pectin), xylan (Xylan), galactose (Galactose), lactose (Lactose), or glycerol (Glycerol) at a final concentration of 0.5% (w:v).

The chemotaxis assay indicated that the effects of attracting SQY 162 differed with root exudates and root extracts. The population density of SQY 162 attracted in the syringe in the KL treatments was higher than that in the K treatments (Fig 1). The attracted population of SQY 162 in the KL treatments was 6.20 log cfu/ml, whereas the attracted number of SQY 162 in the K treatments was 6.03 log cfu/ml. The population densities of SQY 162 in treatments amended with individual carbohydrates were all significantly higher than the control (Fig 2), indicating that all the carbohydrates could efficiently attract the strain. SQY 162 was strongly attracted by sucrose, with as many as 6.62 log cfu/ml observed in the syringe.

### Biofilm assay

**Fig 3:**
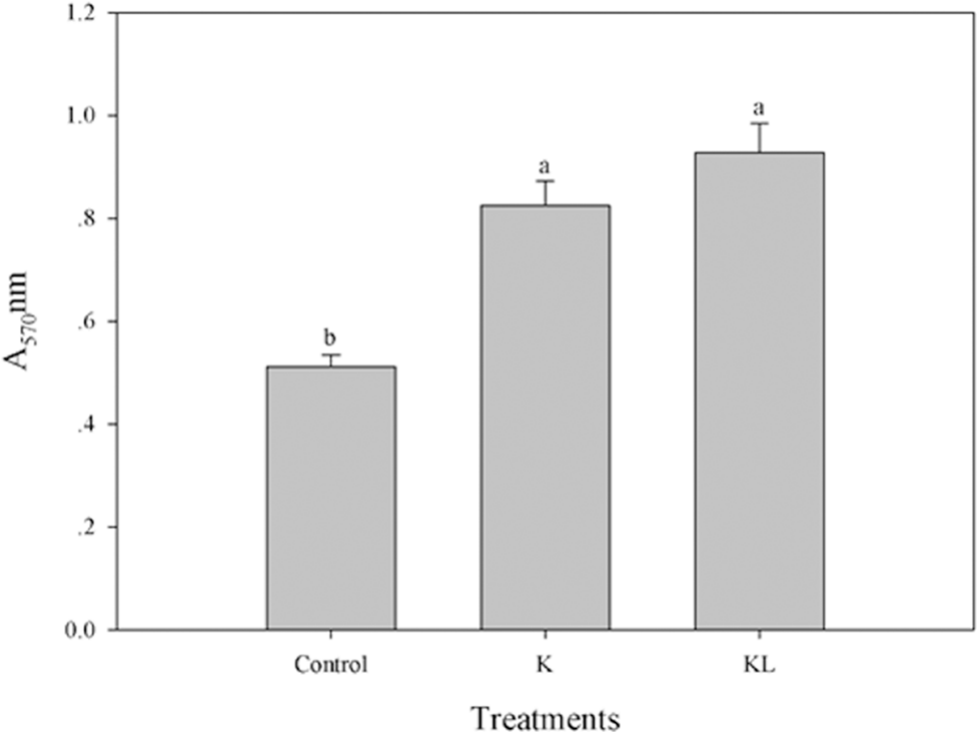
Effects of tobacco root exudates and root extracts from K326 on the biofilm formation of SQY 162. The assays were performed in triplicate and quantified by measuring A_570_ of crystal violet-stained wells rinsed with 80% ethanol and 20% acetone. Control (amended with nothing), K (amended with 15 × root exudates from K326), KL (inoculated with 15 × root extracts from K326).

**Fig 4:**
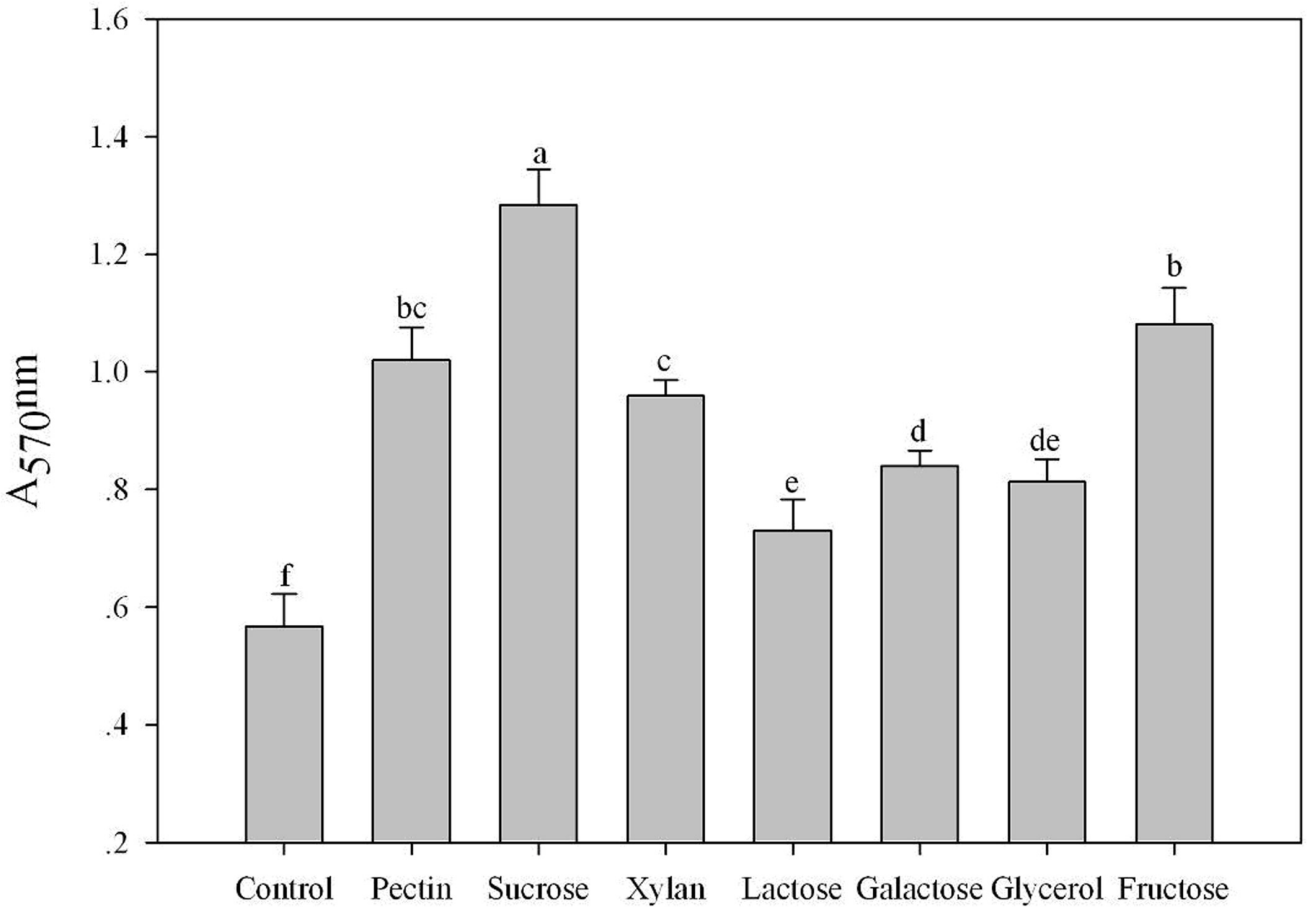
Effects of different carbohydrates on the biofilm formation of SQY 162. The assays were performed in triplicate and quantified by measuring A_570_ nm of crystal violet-stained wells rinsed with 80% ethanol and 20% acetone. The different letters above each bar refer to the Duncan’s test, *p* < 0.05. Control (amended with nothing). LB medium was amended with sucrose (Sucrose), fructose (Fructose), pectin (Pectin), xylan (Xylan), galactose (Galactose), lactose (Lactose), or glycerol (Glycerol) at a final concentration of 0.5% (w:v).

The effects of tobacco root exudates and root extracts on the biofilm formation of SQY 162 are determined. The results indicated that both root exudates and root extracts could induce the biofilm formation of SQY 162 (Fig 3). The effects of carbohydrates on the biofilm formation of SQY 162 were tested using the crystal violet staining method. All the individual carbohydrates induced the biofilm formation of SQY 162, whereas their influence abilities differed (Fig 4). The highest biofilm intensity of SQY 162 was observed in the presence of sucrose, followed by fructose and pectin.

### Effects of carbohydrates on the LPs production

The effects of carbohydrates on the crude LPs production of SQY 162 were summarized in Table 1. Seven carbohydrates, except xylan and glycerol, could induce the production of LPs. In the presence of sucrose, the amount of crude LPs increased to 592.7 mg/L compared to the control. The lowest LPs level, 261.0 mg/L, was observed in the glycerol treatment. Using HPLC analysis, two carbohydrates, pectin and sucrose, induced the production of surfactin (Table 2). However, the five other carbohydrates exhibited little effect on the production of surfactin.

**Table 1:** Effects of different carbohydrate treatments on the lipopeptide production of antagonistic strain SQY 162. Control (amended with nothing).

Treatments	Lipopeptide (mg/L)
Control	295.6±24.4de
Sucrose	592.7±27.2a
Fructose	323.5±43.7cd
Pectin	490.3±16.6b
Xylan	277.0±18.5de
Galactose	359.3±13.5c
Lactose	303.5±28.1de
Glycerol	261.0±17.7e

LB medium amended with sucrose (Sucrose), fructose (Fructose), pectin (Pectin), xylan (Xylan), galactose (Galactose), lactose (Lactose), and glycerol (Glycerol) at a final concentration of 0.5% (w:v).

**Table 2:** Effects of different carbohydrate treatments on the surfactin and Iturin A production of antagonistic strain SQY 162. Control (amended with nothing).

Treatments	Lipopeptides (mg/L)	
	Surfactin	Iturin A
Control	26.29±2.23c	2.69±0.13c
Sucrose	42.40±4.83b	14.38±0.45a
Fructose	27.99±2.16c	2.95±0.10c
Pectin	62.60±7.96a	3.86±0.13b
Xylan	24.23±2.34c	1.85±0.16e
Galactose	24.06±2.18c	1.78±0.11e
Lactose	26.44±1.93c	2.21±0.07d
Glycerol	23.29±1.37c	2.10±0.06de

LB medium amended with sucrose (Sucrose), fructose (Fructose), pectin (Pectin), xylan (Xylan), galactose (Galactose), lactose (Lactose), and glycerol (Glycerol) at a final concentration of 0.5% (w:v).

### Effects of carbohydrates on the growth of SQY 162 and *R*. *solanacearum*

**Fig 5:**
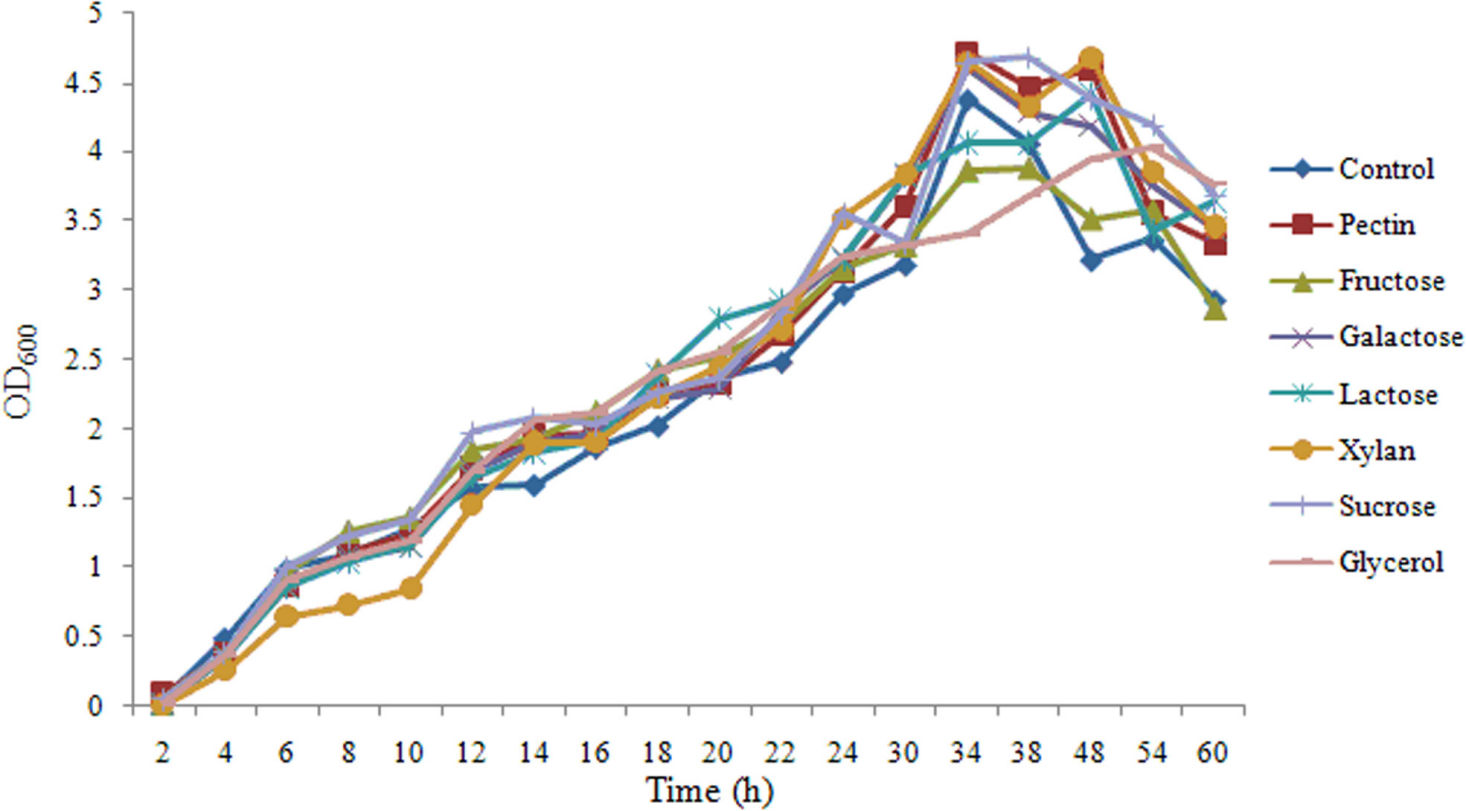
Effects of different carbohydrate treatments on the growth of SQY 162. Control (amended with nothing). LB medium was amended with pectin (Pectin), fructose (Fructose), galactose (Galactose), lactose (Lactose), xylan (Xylan), sucrose (Sucrose), or glycerol (Glycerol) at a final concentration of 0.5% (w:v).

**Fig 6:**
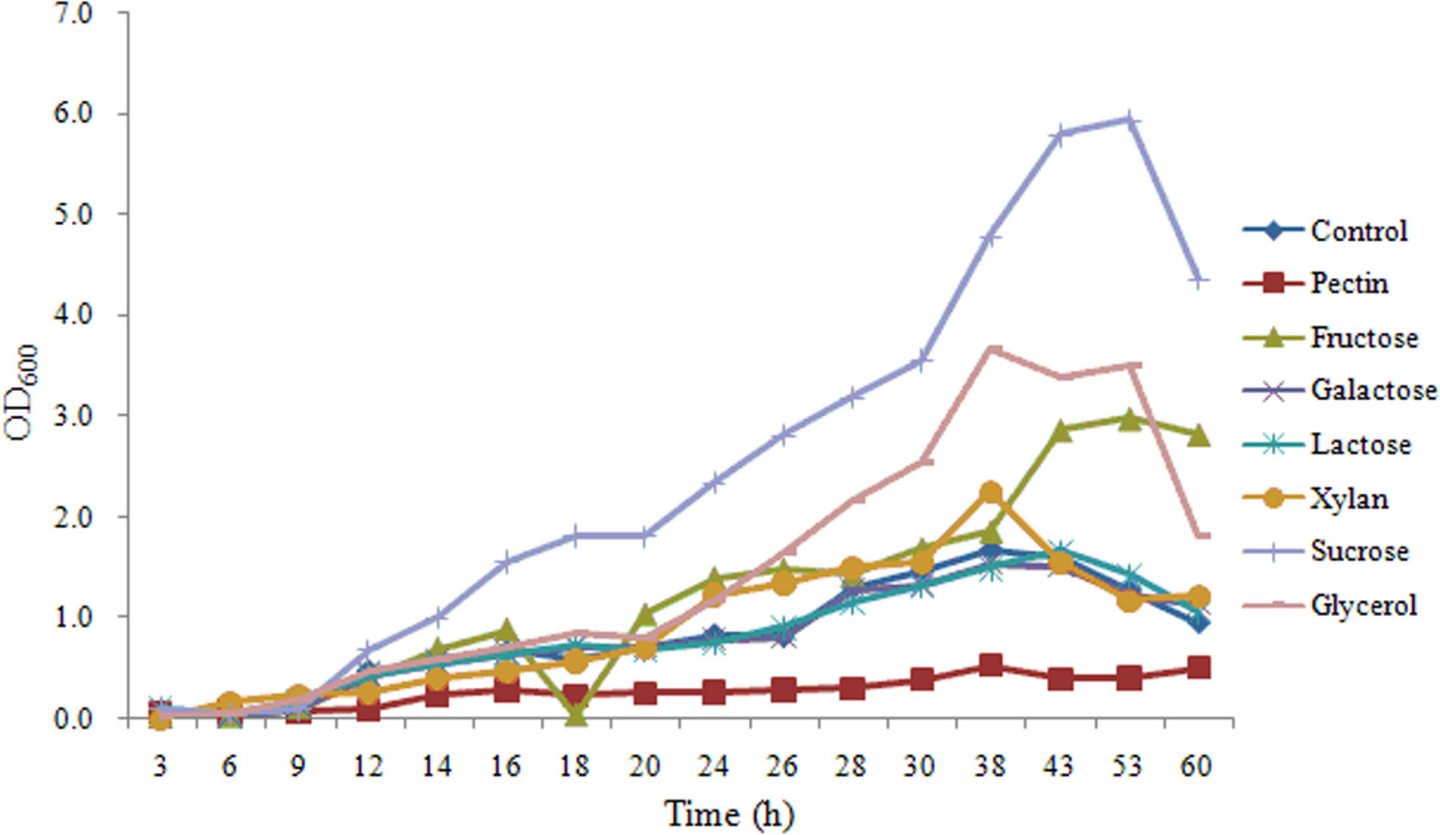
Effects of different carbohydrate treatments on the growth of *R*. *solanacearum*. Control (amended with nothing). Nutrient medium was amended with pectin (Pectin), fructose (Fructose), galactose (Galactose), lactose (Lactose), xylan (Xylan), sucrose (Sucrose), or glycerol (Glycerol) at a final concentration of 0.5% (w:v).

As shown in Fig 5, there were no significant effects of carbohydrates on the growth of SQY 162, indicating carbohydrates might act as induced factors to promote lipopeptide production. For *R*. *solanacearum*, the growth rates of *R*. *solanacearum* in presence of different carbohydrates were different, indicating *R*. *solanacearum* could not equally utilize these carbohydrates (Fig 6).

### Effects of sucrose and pectin on the expression of genes involved in biofilm formation

**Fig 7:**
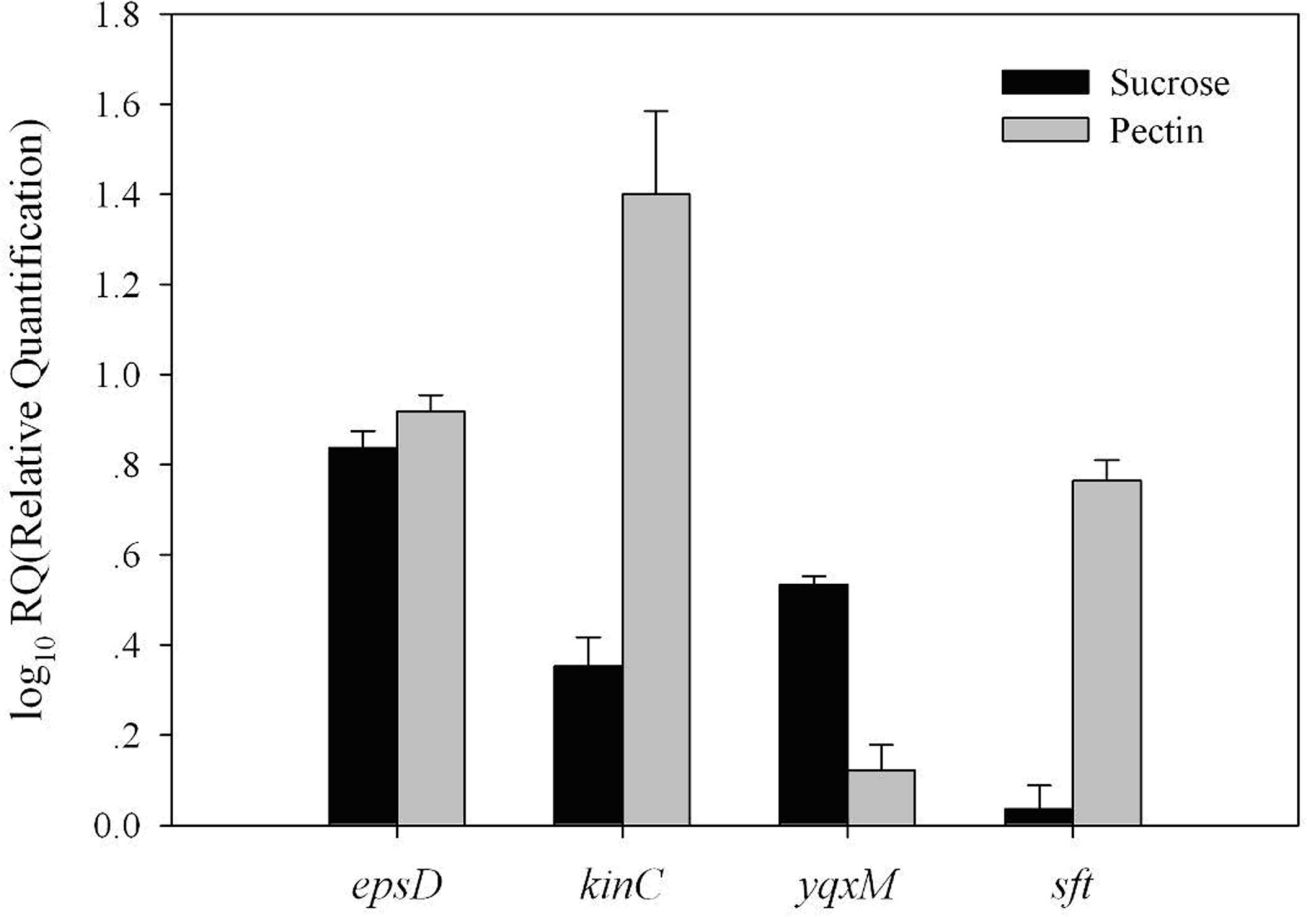
Effects of sucrose or pectin treatments on the transcription levels of biofilm related genes (*eps*D, *kin*C, *yqx*M, and *sft*). LB medium was amended with sucrose (Sucrose) or pectin (Pectin) at a final concentration of 0.5% (w:v).

For the sucrose and pectin treatments, the transcription levels were determined for four genes, *epsD*, *yqxM*, *sft*, and *kinC*, relative to the control. The results suggested that application of sucrose and pectin both significantly increase the transcription levels of *eps*D, *yqx*M and *kin*C in *B*. *amyloliquefaciens* SQY 162. As shown in Fig 7, application of sucrose increased the transcription levels of *eps*D and *yqx*M by 6.89- and 3.43-fold, respectively. However, the *sft* transcription levels showed no significant difference with the addition of sucrose. Furthermore, in contrast to the control, application of pectin significantly increased the transcription levels of *kin*C, *eps*D and *sft* by 26.77-, 8.32- and 5.84-fold, respectively. The results also indicated that addition of pectin had little effect on the *yqx*M transcription levels.

### Pot experiment

**Fig 8:**
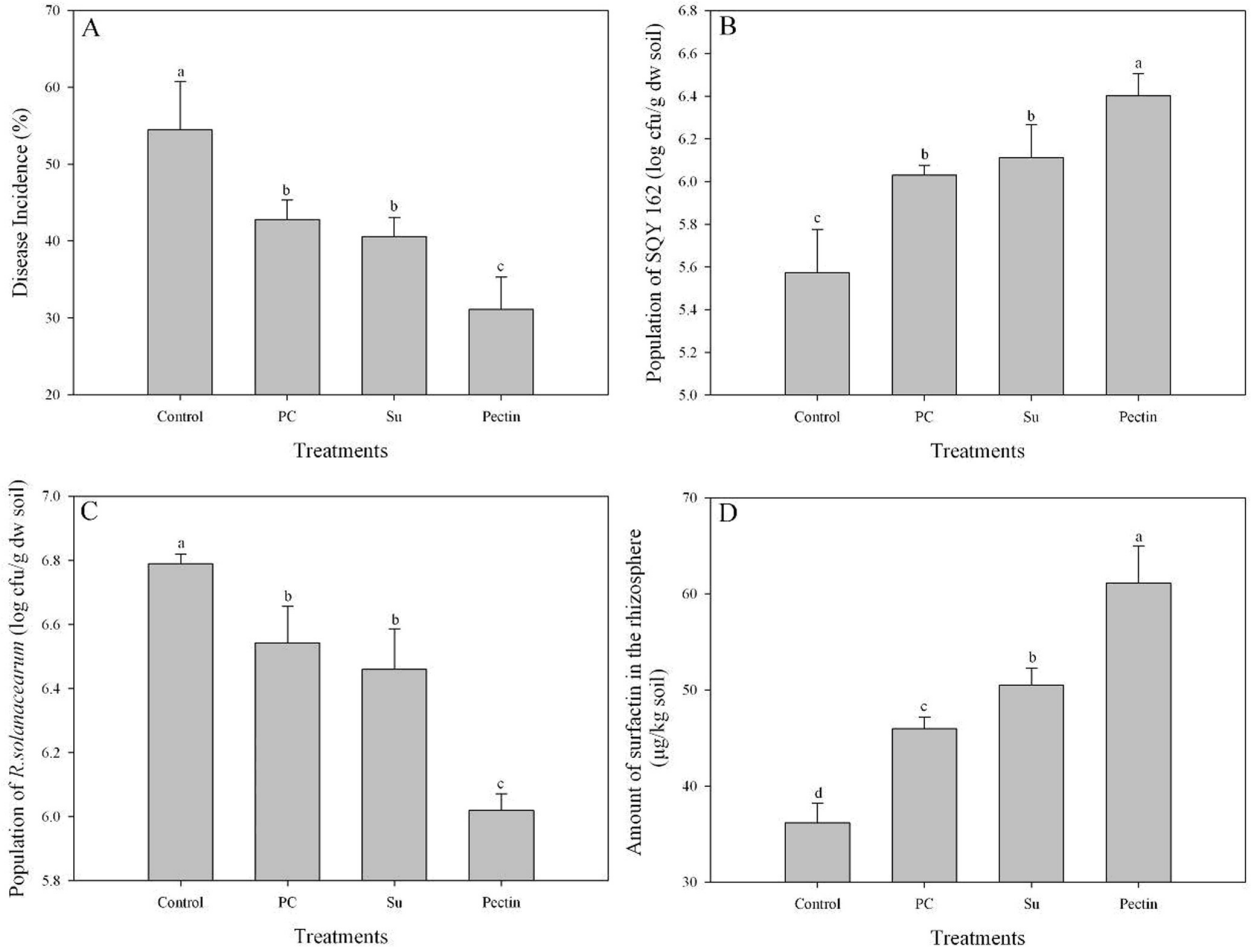
The suppression of tobacco bacterial wilt and quantification of surfactin in pot experiments. Disease incidence, abundance of SQY 162 and *R*. *solanacearum*, and the amount of surfactin were determined in plants 30 days after transplanting. The different letters above each bar refer to the Duncan’s test, *p* < 0.05. Control (soil treated with nothing), PC (yeast extract at a final concentration of 1% was inoculated into each cup), Su (sucrose at a final concentration of 1% was inoculated into each cup), and Pectin (pectin at a final concentration of 1% was inoculated into each cup). Each treatment was inoculated with SQY 162 at a final concentration of 10^7^ cfu/g of soil and performed in triplicate.

The results suggested that treatments amended with carbohydrates efficiently suppressed tobacco bacterial wilt compared to the control treatment (Fig 8). Application of sucrose significantly reduced the disease incidence by 25.55% compared to the control treatment (Fig 8A). However, the suppression efficacy in the Su treatment was equivalent to or slightly higher than that in the PC treatment. The results also showed that the disease incidences of the pectin treatment decreased by 27.34 and 42.83% compared to the PC and control treatments, respectively.

Fig 8B shows that the population of the antagonistic strain SQY 162 decreased with plant growth; however, the decrease ratio differed among treatments. Thirty days after transplanting, the number of SQY 162 in the control treatment remained at 5.57 log cfu/g dry weight (log cfu/g dw) soil. With the application of carbon sources, the populations of SQY 162 in the PC, Su and pectin treatments were all higher than that in the control treatment. The highest population of SQY 162 was observed in the pectin treatment (6.40 log cfu/g dw soil), which was nearly ten times higher than the population in the control. Furthermore, the result also revealed a negative correlation between the population of SQY 162 and *R*. *solanacearum* (r = -0.812, *p* < 0.001). The population of *R*. *solanacearum* in the Control treatment was the highest, up to 6.79 log cfu/g dw soil (Fig 8C). The result showed few differences in the population of *R*. *solanacearum* between the PC and Su treatments. However, with the application of pectin, the population of *R*. *solanacearum* was significantly reduced compared to that of the control treatment.

Using HPLC analysis, the amount of surfactin in the control treatment was 36.17 μg/kg rhizosphere soil (Fig 8D). In contrast, the application of all carbon sources tested significantly increased the production of surfactin in the rhizosphere soil. Application of sucrose and pectin both increased the production of surfactin by 9.86 and 32.96%, respectively, compared to the PC treatment. However, iturin A was not detected in the rhizosphere soil of any treatments.

## Discussion

*Bacillus* spp. is a well-known biological control agent that suppresses bacterial and fungal diseases through competition, the secretion of antibiotics, and the induction of plant systemic resistance. Because the colonization of plant roots by *B*. *amyloliquefaciens* displayed striking similarity to the process of biofilm formation *in vitro* [15], the responses of chemotaxis and biofilm involvement in root colonization are prerequisites for successful biological control [33]. In the present study, tobacco root extracts significantly induced the chemotaxis activity and biofilm formation of SQY 162 compared to the root exudates. These findings corroborated the results of Beauregard et al. [15]. Because the carbohydrates acted not only as energy sources but also as environmental cues to induce the EPS production [34–35], it was not surprising to find that all the carbohydrates tested increased the chemotaxis activity and biofilm formation of the strain SQY 162.

Several studies showed that LPs produced by *Bacillus* increased the formation of biofilm or fruiting bodies [36] and are heavily involved in the antagonistic activities against pathogens *in vitro* [6,37]. In response to different substrates, the production of surfactin and other LPs may be diverse at different levels [38–39]. In the present study, surfactin and iturin were detected in the *B*. *amyloliquefaciens* SQY 162 culture. Pectin and sucrose treatments significantly increased the surfactin and iturin production, resulting in the increase of biofilm biomass. Moreover, analysis of the transcription levels revealed that the increasing production of LPs by the addition of pectin and sucrose were related to the transcriptional stimulation of *sft*, which is involved in surfactin production.

A previous study demonstrated that a *B*. *subtilis* mutant unable to produce surfactin would fail to colonize the root [37]. In addition, surfactin induces potassium leakage, which stimulates the activity of a membrane protein kinase (KinC) and thereby increases expression of the genes involved in matrix synthesis [40]. Due to the stimulation of *Kin*C, addition of pectin could significantly increase the transcription levels of *eps*D and *yqx*M, triggering biofilm formation.

Recent studies have indicated that organic acids significantly increase LPs production in the rhizosphere, resulting in a population increase of *B*. *amyloliquefaciens* S499 on the root [23]. Here, the results of the pot experiment showed that the amount of surfactin in the pectin treatments was significantly higher than that in the control and PC treatments, which was possibly because transcription of *sft* was stimulated by the addition of pectin, as described above. Several studies revealed that inconsistency in bio-control of plant disease by bacterial inoculants in the field could be the result of poor root colonization and insufficient production of antimicrobial metabolites [9,41]. In the present study, we suggested that the bio-control activity of SQY 162 against tobacco bacterial wilt could be enhanced in the presence of pectin. There are several possible mechanisms of the bio-control of the pathogen in the presence of pectin, like enhanced production of surfactin. Since recent studies suggested that surfactin produced by *B*. *amyloliquefaciens* FZB 42 might be most important in colonizing plant roots [22] and the antibiotic effects of the surfactin on *R*. *solanacearum* [42], enhanced production of surfactin in the presence of pectin could reduce the population of the pathogen at the rhizosphere. In contrast, iturin A was not detected in the rhizosphere, mainly due to the detection limits for LPs from the soil extracts [43]. It was also found that the fengycin and iturin with antifungal activities produced by *B*. *amyloliquefaciens* S499 were both poorly expressed *in planta*. Therefore, whether surfactin could be expressed in plant needs further study. Enhanced production of surfactin by pectin amendment could enable the bacterium to successful colonize the highly competitive rhizosphere niche. It was suggested that an inoculant strain with high rhizosphere competence ability is a key factor for successful disease control [44]. The addition of pectin and sucrose both significantly increased the population of SQY 162 in the rhizosphere, which decreased the pathogen population to some degree. Furthermore, surfactin produced by *B*. *subtilis* may trigger induced systemic resistance to protect the plant from pathogen infection [45]. It was also suggested oligogalacturonides released by bacterial pectinases could also induce plant defense [46]. Interestingly, *R*. *solanacearum* was found to grow slower in nutrient medium in the presence of pectin than other carbohydrates amendment in present study, reducing the disease incidence in some aspects. However, *R*. *solanacearum* was found to utilize pectin as carbon source with pectinase [47]. Therefore, another interest was to study whether the plant extracts could affect the biofilm and colonization of *R*. *solanacearum* on the root.

In conclusion, our results suggest that plant root extracts from tobacco supply nutrient sources for bacterial growth in the rhizosphere, and induce chemotaxis and biofilm formation. Addition of pectin decreased the abundance of *R*. *solanacearum* and disease incidence of tobacco bacterial wilt by inducing antibiotic secretion and SQY 162 colonization in the rhizosphere. Although many factors in the rhizosphere affect antibiotic secretion and *Bacillus* colonization, these results increase our understanding of optimizing bio-control strategies using this strain.

## Supporting Information

S1 Fig Antibiotic activity of SQY 162 against the pathogen *in vitro*. (TIF)
