# Supplementary figures and images for: Pectin Enhances Bio-Control Efficacy by Inducing Colonization and Secretion of Secondary Metabolites by Bacillus amyloliquefaciens SQY 162 in the Rhizosphere of Tobacco
Source: PLoS One. 2015 May 21;10(5):e0127418. doi: 10.1371/journal.pone.0127418 (PMC4440637; doi:10.1371/journal.pone.0127418)

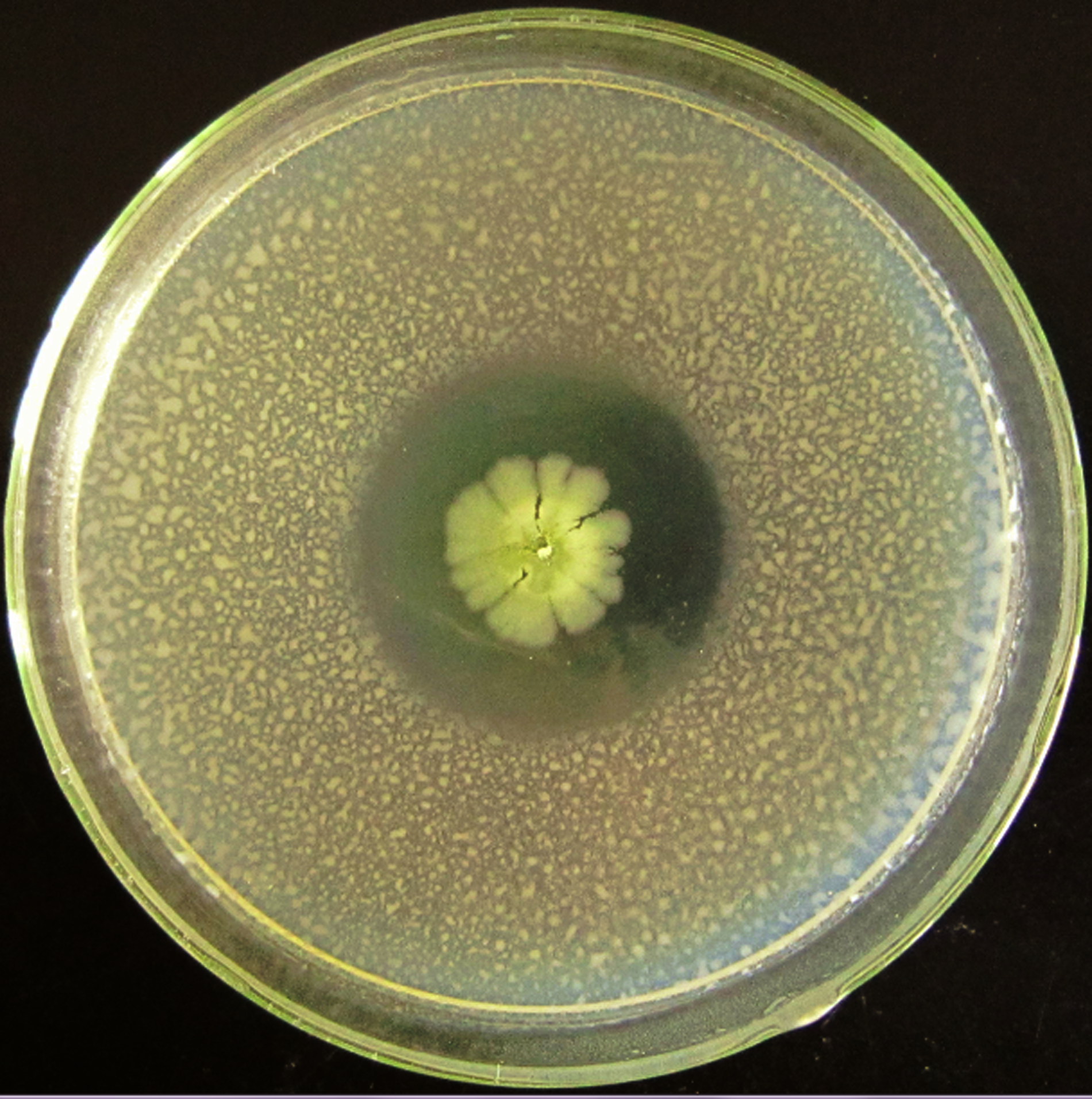

Supplement: S1 Fig — (TIF) [file pone.0127418.s001.tif]
